# Development of a Conversion Table Linking Functional Independence Measure Scores to International Classification of Functioning, Disability, and Health Qualifiers: Insights from a Survey of Healthcare Professionals

**DOI:** 10.3390/healthcare12080831

**Published:** 2024-04-15

**Authors:** Shu Umemori, Mao Ogawa, Shin Yamada, Masayo Komatsu, Emiko Oikawa, Yasuyo Okamoto, Masaki Katoh, Tomohide Shirasaka, Kagari Abiko, Shigehiro Moriizumi, Yuichiro Matsuo, Harukazu Tohyama, Masahiko Mukaino

**Affiliations:** 1 Department of Rehabilitation Medicine, Hokkaido University Hospital, Sapporo 060-8648, Japan; s.ummr@huhp.hokudai.ac.jp (S.U.); maogw@huhp.hokudai.ac.jp (M.O.); 2 Department of Rehabilitation Medicine, Sapporo Azabu Neurosurgical Hospital, Sapporo 065-0022, Japan; 3 Department of Rehabilitation Medicine, Kyorin University School of Medicine, Mitaka 181-8611, Japan; yamada-shin@ks.kyorin-u.ac.jp; 4 Division of Environmental Medicine and Population Sciences, Department of Social Medicine, Graduate School of Medicine, Osaka University, Suita 565-0871, Japan; kmasayo@envi.med.osaka-u.ac.jp; 5 General Incorporated Association, Japan ICF Association, Tama 206-0012, Japan; icfeverybody@gmail.com; 6 Department of Rehabilitation, Hanakawa Hospital, Ishikari 061-3207, Japan; y.okamoto@kyouseikai.jp; 7 Department of Rehabilitation, Fujita Health University Hospital, Toyoake 470-1192, Japan; katomasa@fujita-hu.ac.jp; 8 Department of Rehabilitation Medicine, Hokuto Social Medical Corporation Tokachi Rehabilitation Center, Obihiro 080-0833, Japan; shirasaka@hokkaido.med.or.jp; 9 Department of Rehabilitation Medicine, Moriyama Memorial Hospital, Asahikawa 070-0832, Japan; moriko@mail.goo.ne.jp; 10 Department of Rehabilitation Medicine, National Hospital Organization Hokkaido Medical Center, Sapporo 063-0005, Japan; matsuo.yuichiro.mq@mail.hosp.go.jp; 11 Faculty of Health Sciences, Hokkaido University, Sapporo 060-0812, Japan; tohyama@med.hokudai.ac.jp

**Keywords:** ICF, qualifiers, functioning

## Abstract

In clinical practice, patient assessments rely on established scales. Integrating data from these scales into the International Classification of Functioning, Disability, and Health (ICF) framework has been suggested; however, a standardized approach is lacking. Herein, we tested a new approach to develop a conversion table translating clinical scale scores into ICF qualifiers based on a clinician survey. The survey queried rehabilitation professionals about which functional independence measure (FIM) item scores (1–7) corresponded to the ICF qualifiers (0–4). A total of 458 rehabilitation professionals participated. The survey findings indicated a general consensus on the equivalence of FIM scores with ICF qualifiers. The median value for each item remained consistent across all item groups. Specifically, FIM 1 had a median value of 4; FIM 2 and 3 both had median values of 3; FIM 4 and 5 both had median values of 2; FIM 6 had a median value of 1; and FIM 7 had a median value of 0. Despite limitations due to the irreconcilable differences between the frameworks of existing scales and the ICF, these results underline the ICF’s potential to serve as a central hub for integrating clinical data from various scales.

## 1. Introduction

The International Classification of Functioning, Disability, and Health (ICF) is a comprehensive framework for assessing health status, encompassing body function, structure, activity, and participation, as well as the environmental and individual factors influencing these elements. In addition to offering an exhaustive set of categories for human functioning, it incorporates qualifiers that function as scale systems to delineate the severity of problems. However, despite their design for use in international statistics, the actual implementation of ICF in real-world settings has encountered substantial obstacles. Key challenges in this implementation include the vast number of categories, the intricate nature of category definitions, and the inconsistent reliability of ratings. Efforts to overcome these barriers have involved the development of disease-specific core sets, simplification of item definitions, and provision of reference guides for assessment [1,2,3].

In clinical practice, the assessment of functioning information often relies on a variety of established assessment scales, such as the Barthel Index [4] and the Functional Independence Measure (FIM) [5], which are commonly used to evaluate activities of daily living (ADL), as well as disease-specific scales such as the National Institutes of Health Stroke Scale (NIHSS) for stroke patients [6]. While there have been significant efforts to adapt the ICF for clinical practice, the complete replacement of existing, widely used scales with the ICF remains a significant challenge, considering their established utility. In this regard, linking the data from existing scales to the ICF may be an alternative solution. In this context, Cieza et al. proposed a ‘linking rule’ to integrate data from existing scales into the ICF framework [7,8,9]. This approach, which has seen numerous applications [10,11], suggests the potential of consolidating information from various clinical scales into the ICF. Such integration could enhance the comparability of functioning information across the different clinical contexts where various scales are employed. While information from different scales cannot be directly compared with absolute accuracy, consolidating and mapping a wide array of clinical information within the ICF framework could significantly improve our understanding of the relationship between diseases and functioning statuses. However, a standardized method for incorporating scores from existing clinical scales into the ICF has not yet been established.

To address this gap and explore a novel approach to solving the issue, we aim to develop a conversion table that translates clinical scale scores into ICF qualifiers. In this study, we set FIM, which is widely used in rehabilitative clinical practice, as a first target. The FIM, a clinician-rated scale familiar to most rehabilitation clinicians, potentially facilitates easier consensus formation than patient questionnaires, which necessitate a more in-depth consideration of patient perceptions. With its multi-level 1–7 rating options for each item and clear scoring guidelines, the FIM is well-suited for data conversion into the 5-point scale of ICF qualifiers, especially when compared to scales offering fewer rating options, like binary scales. This study employed clinician surveys as its methodology, with the objective of eliciting insights from rehabilitation professionals on how to align FIM rating options with ICF qualifiers. Specifically, a survey was conducted using a structured questionnaire designed to explore clinicians’ perceptions of the relationship between FIM ratings and ICF qualifiers. Based on the insights gathered, a proposal for a conversion table was subsequently developed.

## 2. Materials and Methods

### 2.1. Participants

Clinician survey: Rehabilitation professionals, including physiatrists (MDs), physical therapists (PTs), occupational therapists (OTs), speech therapists (STs), and social workers (SWs), were invited to participate in this survey. To ensure the reliability and validity of the survey, the survey was planned to collect from multiple sites and to satisfy the required sample size. The survey was carried out across seven hospitals in Japan. With a margin of error of 5% and a confidence level of 95%, the calculated required sample size was defined as 384 individuals [12]. An online survey was created using Google Forms, and the link was distributed to 657 rehabilitation professionals working at the seven hospitals. A total of 458 individuals (response rate: 69.7%) participated in the study. The mean age of participants was 31.1 ± 8.0 years, ranging from 22 to 64 years, with 252 males and 206 females. The professional backgrounds of the respondents included 4 physiatrists, 225 physical therapists, 166 occupational therapists, 62 speech–language pathologists, and 1 social worker.

Expert review: Six experienced rehabilitation professionals, with expertise in ICF research, participated in the review process. The participant group comprised 2 physiatrists, 2 physical therapists, 1 occupational therapist, and 1 nurse.

### 2.2. Item Linking Table

Previously, the item-linking table between the FIM and ICF was developed by the ICF Implementation Working Group (set 2019–2021), established under the Functional Classification Expert Committee as part of the Statistics Subcommittee of the Social Security Council of the Japanese Ministry of Health, Labor, and Welfare (Appendix A, [13]). Briefly, the consensus process involved three steps. First, four clinical experts familiar with the ICF were asked to link FIM items and ICF entities. The experts were then asked to refer to the linking rules [7,8,9], and to follow the additional rules to simplify the subsequent scale-linking process: (1) the scale items should be linked to one major entity that is most relevant, and (2) the entity linked should be a second-level category, instead of third or fourth entities, if possible. Based on the linking results, the experts discussed forming a consensus if there was a disagreement. The linking table was then finalized, as shown in Appendix A.

### 2.3. Survey for Linking Scores and Qualifiers

A survey was conducted using a questionnaire asking whether a score of 1–7 on the FIM (version 3) items would fall into the ICF 0–4 qualifiers.

To avoid excessive complexity with varied score linking results, the items were grouped based on the chapters of the linked ICF categories: b1—mental functions; b5—functions of the digestive, metabolic, and endocrine systems; b6—genitourinary and reproductive functions; d1—learning and applying knowledge; d3—communication; d4—mobility; d5—self-care; d7—interpersonal interactions and relationships. Then, the “Sphincter control” items, including bladder and bowel control, belonging Chapters b5 and b6, were grouped into one, as these have very similar rating standards. Finally, a survey was conducted on seven groups of FIM items. For each item group, participants were asked to categorize scores ranging from 1 to 7 into specific ICF qualifiers: 0 indicating “No problem”, 1 for “Mild problem”, 2 for “Moderate problem”, 3 for “Severe problem”, and 4 for “Complete problem”. Participants were asked to consult the original ICF coding guidelines, as found in Annex 2 in the ICF [14]. Japanese guidance formulated by the ICF Implementation Working Group, which is based on the original coding guidelines, was also provided as a reference [13].

### 2.4. Data Analysis and Development of a Conversion Table

After the data were collected, we calculated the response ratios for the corresponding ICF qualifiers for each FIM score across the item groups. The median, mean, and standard deviation for the patients’ responses were also determined. A draft conversion table was then constructed to map FIM scores to ICF qualifiers. Subsequently, an expert review was conducted involving a panel of rehabilitation professionals well-versed in the ICF. This step aimed to confirm the content validity of the conversion table and identify any discrepancies with the ICF coding guidelines.

## 3. Results

The ratios of the answers are shown in Table 1. In the survey, the most common responses regarding the equivalent ICF qualifiers for each option of the FIM were as follows: for the self-care items, the most frequent responses for the FIM scores of 1, 2, 3, 4, 5, 6, and 7 were 4 (97.6%), 3 (79.1%), 3 (57.6%), 2 (91.3%), 2 (71.8%), 1 (92.8%), and 0 (99.1%); for the mobility/transfer items, they were 4 (97.1%), 3 (79.4%), 3 (58.3%), 2 (91.2%), 2 (70.3%), 1 (92.5%), and 0 (99.1%); for the bladder/bowel management items, they were 4 (96.9%), 3 (81.2%), 3 (55.0%), 2 (88.5%), 2 (52.1%), 1 (83.4%), and 0 (98.9%); for the communication items, they were 4 (97.1%), 3 (84.4%), 3 (55.1%), 2 (90.8%), 2 (53.8%), 1 (88.2%), and 0 (98.9%); for the solving problems items, they were 4 (96.8%), 3 (84.1%), 3 (53.0%), 2 (91.8%), 2 (50.5%), 1 (86.6%), and 0 (99.0%); for the social interaction items, they were 4 (98.1%), 3 (87.8%), 3 (55.7%), 2 (87.6%), 2 (50.9%), 1 (82.3%), and 0 (99.2%); for the memory items, they were 4 (98.2%), 3 (86.7%), 3 (58.8%), 2 (89.8%), 2 (51.2%), 1 (83.1%), and 0 (99.2%). The median values for each item were consistent across all item groups, as follows: FIM 1 corresponded to ICF 4, FIM 2 and 3 were equivalent to ICF 3, FIM 4 and 5 matched ICF 2, FIM 6 aligned with ICF 1, and FIM 7 was equal to ICF 0. When considering the average ICF values for each FIM score (FIM 1–7), a slight variation was found between items. For example, the average ICF value for FIM 5 was 1.7 for both the self-care and mobility/transfer items, whereas it was 1.5 for the bladder/bowel management, communication, solving problems, social interaction, and memory items.

A draft conversion table was developed based on the median and mean values derived from the survey; this was then subjected to expert review. Since the mean values across all item groups were consistent, a singular conversion table mapping FIM scores to ICF qualifiers was created. However, during the expert review, the version of the table that relied on mean values was dismissed due to inconsistencies with the underlying concepts of ICF qualifiers and concerns regarding scientific validity. The conversion table that ultimately received endorsement is presented in Table 2.

## 4. Discussion

In this study, we conducted a survey of 458 clinical experts to explore their perspectives on the interrelationships between FIM items and ICF qualifiers. The survey covered FIM items related to self-care, mobility/transfer, voiding control, communication, problem solving, social interaction, and memory. Despite some variation in responses, the median ICF qualifier remained homogenous across different items, yielding the following translations of ICF qualifiers: 4 for FIM 1, 3 for FIMs 2 and 3, 2 for FIMs 4 and 5, 1 for FIM 6, and 0 for FIM 7.

The response distribution for FIM 1, 2, 4, 6, and 7 showed a high level of agreement among over three-quarters of the participants. In contrast, FIM 3 and 5 elicited a wider range of responses. Specifically, for FIM 3, the majority, ranging from 53.0% to 58.8%, believed that it merited a score of 3 in the ICF, whereas 40.4% to 46.3% felt that it deserved a score of 2. Regarding FIM 5, 50.5% to 71.8% of the respondents assigned a score of 2 in the ICF, while 27.3% to 49.3% considered a score of 1 to be more appropriate. The inequality of rating standards in their definitions may have affected this variation. For example, in the FIM, a score of 4 represents mild problems or the subjects expends 50–74% of effort [5], while the qualifier of 3 in ICF, which was most frequent answer equivalent to FIM 4, is defined in the Annex 2 coding guideline of ICF [14] as a “moderate problem (50–95%)”. Additionally, the scope of the items under consideration could influence the level of agreement. For example, when evaluating the task of dressing, the FIM focuses primarily on basic actions like “taking off” and “putting on”, without considering the degree of assistance required for preparing and storing clothes. In contrast, the ICF’s definition of dressing encompasses broader aspects, including the choice of clothing appropriate for different situations. These differences in the definitions of rating standards and the scope of the items could account for the observed variation in responses to FIM 3. Further, the score of 5 in FIM, signifying supervision [5], predominantly equated to a score of 2 in the ICF qualifiers, which indicates a moderate problem with “up to half of the scale of total difficulty” [14]. However, it remains notable that, in some item groups, nearly half of the participants considered this to be equivalent to 1 in the ICF qualifiers, defining “5% to 24%” of the problem, which may depend on how difficult it is for the patient to conduct daily activities under supervision. An activity performed under supervision may be considered a mild problem as it does not require manual assistance, while in some cases, it can be a significant problem as the individual needs someone present when performing the given activity. While the rating in FIM is focused on independence in the activity, the ICF aims to integrate broader aspects of patients’ experiences in capturing functioning problems, and this difference in concept might also affect the variety in responses of the participants.

While responses exhibited some variability, the process of abstracting functional information from existing scales and aligning it with the common framework of the ICF offers significant merit. Currently, various clinical rating scales are used for assessing functioning for the patients with different diseases and health conditions and for different purposes. For example, for stroke patients, NIHSS [6] is frequently used; for patients with Parkinson’s disease, the Unified Parkinson’s Disease Rating Scale developed by Movement Disorder Society (MDS-UPDRS) [15] is used; for patients with spinal cord injury, International Standards for Neurological Classification of Spinal Cord Injury (ISNCSCI) [16] and the Spinal Cord Independence Measure (SCIM) [17] are used; FIM and Barthel Index are used across various diseases but with a specific focus on ADL. However, in terms of international statistics, the diversity of these clinical scales makes it difficult to achieve comparability between assessments. While several studies have investigated the relationships between functioning rating scales [18,19,20], they have frequently concentrated on a restricted set of scales, such as the FIM, BI, and modified Rankin Scale (mRS), for comparison. Furthermore, these comparisons were typically based on the total scores of the scales rather than on item-by-item analysis. Although these studies are valuable for facilitating comparisons of functioning information across different contexts, they are insufficient in providing a comprehensive comparison of functioning information in clinical contexts, where a broader spectrum of clinical scales are used. The ICF, which encompasses a comprehensive classification framework for functioning, including ADL, could potentially serve as a central hub for integrating clinical information, including extensive numbers of clinical scales. Indeed, numerous studies have been conducted to link items from clinical scales to the ICF [21,22,23]; however, a consensus on how we can accurately reflect the severity of problems measured by these scales in the ICF framework remains elusive. There have been several initiatives to map existing clinical scales to the ICF qualifiers. For instance, the approach of utilizing expert panels for proposing integration solutions has been considered [24,25]. This method is quite practical, though it encounters some limitations in terms of broad applicability. Specifically, when these discussions are held within smaller groups, there is a need to ensure that the proposed solutions can be generalized in clinical practice. In contrast, some studies have taken a more rigorous approach by collecting clinical data [26,27]. This approach is scientifically robust, allowing for more precise conversion to be conducted. Furthermore, gathering clinical data contributes valuable statistical insights, including the generation of interval metrics derived from the data. However, a considerable amount of effort and resources are required for the collection and detailed analysis of clinical data. Despite the effectiveness of these established methods, there remains an opportunity to investigate other approaches that could offer new solutions.

The methodology suggested in this study, which involves the creation of a conversion table derived from clinician survey interpretations, could offer another practical solution to the issue of integrating clinical scales with the ICF. The survey results unexpectedly aligned into a simple conversion table based on the median ICF scores. The ICF qualifiers correspond to the FIM scores in the following manner: a qualifier of 4 for FIM 1; a qualifier of 3 for FIMs 2 and 3; a qualifier of 2 for FIMs 4 and 5; a qualifier of 1 for FIM 6; and a qualifier of 0 for FIM 7. This method is both generalizable and easy to implement, aiding in the alignment of various clinical scales with the ICF. It offers clear and concise mapping of the relationship between clinical scales and ICF scores. While it may not match the rigor of more complex approaches, this method could be beneficial in consolidating a wide range of clinical data for broader applications in international statistics.

This study had several limitations. First, some of the items, such as self-care, included multiple sub-items such as eating, dressing, and changing clothes, and the interrelationship between the FIM and ICF for each of these items was not investigated separately. This approach was adopted to simplify the survey, with the aim of collecting a larger number of participants. While a more detailed questionnaire could potentially highlight differences between categories, the uniformity of results across item groups supports the robustness of our findings. This consistency suggests that the overall results might not be significantly impacted by this limitation. Second, as previously mentioned, the FIM and ICF do not perfectly align in their rating standards and assessment scope. Consequently, the conversion table may not be suitable for individual data analyses. This method is more appropriate for large-scale studies, such as those based on population samples, in which broader trends and patterns are the focus. Third, the study does not include testing with real-world examples. While the validity of the conversion table is reinforced through expert review, its applicability and effectiveness in actual clinical practice remain to be further validated using real-world data. Additionally, since the study was conducted solely in Japan, the generalizability of the results to other contexts warrants further examination. Finally, the conversion table based on the median values reduces the information from the FIM. Using averaged values may retain more granularity, but this approach may not be permissible for statistical purposes as the FIM is an ordinal scale. Indeed, this method was conclusively dismissed during the expert review. Further research could be valuable in uncovering a range of practical methodologies for data conversion, which might differ based on the specific goals of the analysis.

## 5. Conclusions

In this study, a survey was conducted to investigate rehabilitation clinicians’ interpretations of the relationship between the FIM and ICF qualifier scores. Overall, the survey results revealed an interrelationship between the FIM scores and the ICF qualifiers, leading to the development of a conversion table. Further investigation will be beneficial for uncovering practical methods for data conversion.

## Figures and Tables

**Table 1:** Percentage distribution of participants across equivalent ICF qualifiers for each FIM response option.

		Included FIM Scores						
Item Groups	Qualifiers	1	2	3	4	5	6	7
Self-Care	0	0.2	0.2	0.2	0.2	0.4	6.6	99.1
	1	0.0	0.0	0.4	4.6	27.3	92.8	0.2
	2	0.9	1.1	40.4	91.3	71.8	0.4	0.7
	3	1.3	79.7	57.6	3.9	0.4	0.2	0.0
	4	97.6	19.0	1.3	0.0	0.0	0.0	0.0
	Median	4	3	3	2	2	1	0
	Mean (SD)	4.0 (0.3)	3.2 (0.4)	2.6 (0.5)	2.0 (0.3)	1.7 (0.5)	0.9 (0.3)	0.0 (0.2)
Mobility/Transfer	0	0.2	0.0	0.0	0.2	0.7	6.8	99.1
	1	0.0	0.2	0.7	4.2	28.6	92.5	0.4
	2	0.9	1.3	40.4	91.2	70.3	0.7	0.4
	3	1.8	79.4	58.3	4.4	0.4	0.0	0.0
	4	97.1	19.1	0.7	0.0	0.0	0.0	0.0
	Median	4	3	3	2	2	1	0
	Mean (SD)	4.0 (0.3)	3.2 (0.4)	2.6 (0.5)	2.0 (0.3)	1.7 (0.5)	0.9 (0.3)	0.0 (0.1)
Bladder/Bowel management	0	0.2	0.0	0.0	0.2	0.9	15.7	98.9
	1	0.0	0.2	0.9	6.6	46.8	83.4	0.7
	2	1.1	1.5	43.0	88.5	52.1	0.9	0.4
	3	1.8	81.2	55.0	4.6	0.0	0.0	0.0
	4	96.9	17.0	1.1	0.0	0.0	0.0	0.0
	Median	4	3	3	2	2	1	0
	Mean (SD)	4.0 (0.3)	3.2 (0.4)	2.6 (0.5)	2.0 (0.3)	1.5 (0.5)	0.9 (0.3)	0.0 (0.1)
Communication	0	0.0	0.0	0.0	0.0	0.4	10.3	98.9
	1	0.2	0.2	0.7	5.3	45.1	88.2	0.9
	2	0.7	1.5	43.3	90.8	53.8	1.3	0.2
	3	2.0	84.4	55.1	3.9	0.7	0.2	0.0
	4	97.1	13.8	0.9	0.0	0.0	0.0	0.0
	Median	4	3	3	2	2	1	0
	Mean (SD)	4.0 (0.3)	3.1 (0.4)	2.6 (0.5)	2.0 (0.3)	1.5 (0.5)	0.9 (0.3)	0.0 (0.1)
Solving problems	0	0.2	0.0	0.0	0.0	0.2	12.9	99.0
	1	0.0	0.5	0.2	5.7	49.3	86.6	1.0
	2	0.5	0.5	46.3	91.8	50.5	0.5	0.0
	3	2.5	84.1	53.0	2.5	0.0	0.0	0.0
	4	96.8	14.9	0.5	0.0	0.0	0.0	0.0
	Median	4	3	3	2	2	1	0
	Mean (SD)	4.0 (0.3)	3.1 (0.4)	2.5 (0.5)	2.0 (0.3)	1.5 (0.5)	0.9 (0.3)	0.0 (0.1)
Social interaction	0	0.0	0.0	0.0	0.0	2.1	16.9	99.2
	1	0.3	0.3	0.3	8.5	47.0	82.3	0.5
	2	0.5	0.8	43.5	87.6	50.9	0.5	0.3
	3	1.0	87.8	55.7	3.9	0.0	0.3	0.0
	4	98.1	11.1	0.5	0.0	0.0	0.0	0.0
	Median	4	3	3	2	2	1	0
	Mean (SD)	4.0 (0.2)	3.1 (0.3)	2.6 (0.5)	2.0 (0.3)	1.5 (0.5)	0.8 (0.4)	0.0 (0.1)
Memory	0	0.0	0.0	0.0	0.0	0.5	16.1	99.2
	1	0.3	0.5	0.3	5.6	48.3	83.1	0.3
	2	0.5	0.8	40.4	89.8	51.2	0.8	0.5
	3	1.0	86.7	58.8	4.6	0.0	0.0	0.0
	4	98.2	12.0	0.5	0.0	0.0	0.0	0.0
	Median	4	3	3	2	2	1	0
	Mean (SD)	4.0 (0.2)	3.1 (0.4)	2.6 (0.5)	2.0 (0.3)	1.5 (0.5)	0.8 (0.4)	0.0 (0.2)

**Table 2:** Conversion table for ICF and FIM.

FIM	ICF
1	4—Complete problem
2	3—Severe problem
3	
4	2—Moderate problem
5	
6	1—Mild problem
7	0—No problem

## Data Availability

The data presented in this study are available from the corresponding author upon reasonable request.

## Appendix A

**FIM**		**ICF**
Self-Care	Eating	d550: eating/d560: drinking
	Grooming	d520: caring for body parts
	Bathing	d510: washing oneself
	Dressing—upper	d540: dressing
	Dressing—lower	d540: dressing
Transfers	Bed, chair, wheelchair	d420: transferring oneself
	Toilet	d420: transferring oneself
	Tub, shower	d420: transferring oneself
Locomotion	Walk/Wheelchair	d450: walking/d465: moving around using equipment
	Stairs	d451: going up and down stairs
Sphincter control	Bladder	b620: urination functions
	Bowel	b525: defecation functions
Communication	Comprehension	d310: communicating with receiving spoken messages/d315: communicating with receiving nonverbal messages
	Expression	d330: speaking/d335: producing nonverbal messages
Social cognition	Problem solving	d175: solving problems
	Social interaction	d710: basic interpersonal interactions
	Memory	b144: memory functions
